# Lytic Bone Lesion: An Unusual Presentation of Hairy Cell Leukemia

**DOI:** 10.7759/cureus.12959

**Published:** 2021-01-28

**Authors:** Sydney M Fasulo, Spandana Narvaneni, Vinod Kumar, Anusha Manje Gowda, Yasmeen Sultana

**Affiliations:** 1 Hematology/Oncology, St. Joseph's University Medical Center, Paterson, USA; 2 Internal Medicine, St. Joseph's University Medical Center, Paterson, USA

**Keywords:** massive splenomegaly, hairy cell leukemia, cladribine, moxetumomab pasudotox, rituximab

## Abstract

Hairy cell leukemia (HCL) is a seldom encountered malignancy of lymphocytes with a low incidence in the United States. HCL generally follows an indolent course and not all patients require treatment. Most patients are asymptomatic at the time of diagnosis. Treatment is reserved for those with anemia, thrombocytopenia, neutropenia, recurrent infections, symptomatic splenomegaly, or lymphadenopathy impairing vital organ function. Purine analogs are the mainstay of treatment with a durable response. We report a case of a 49-year old Ukrainian male who presented with bone pain secondary to a lytic bone lesion who was diagnosed with HCL.

## Introduction

Hairy cell leukemia (HCL ) is a rare malignant lymphoproliferative disorder that is often asymptomatic but may present with cytopenia and splenomegaly due to circulating abnormal B-cells. It was first identified by Bouroncle et al. [1] in 1958 and has an estimated incidence, in the United States, of three cases per million persons per year, which equates to approximately 1000 new cases each year [2]. The abnormal B-cells are characterized by a central nucleus, thin cytoplasmic projections resembling hair, and the expression of CD11c, CD25, CD103, CD123, CD20, CD22, CD52, and mild expression of cyclin D [3].

## Case presentation

We report a case of a 49-year old Ukrainian male with a past medical history of HCL treated seven years prior in Ukraine with cladribine chemotherapy. The patient presented to the emergency department with chest, left shoulder, and right hip pain for three weeks duration. He reported two to three months of increasing fatigue leading to progressive weakness over the week prior. He is a life-long non-smoker and consumes minimal alcohol. Denies fever, chills, night sweats, weight loss, or abdominal pain. He moved from Ukraine to the United States one year prior and denied Chernobyl exposure.

In the emergency department, the patient was found to have a temperature of 37.2 degrees Celsius, heart rate of 111 beats/min, blood pressure of 148/88 mmHg, respiratory rate of 16/min. His initial complete blood count demonstrated a white blood cell count of 1.2x109/L (normal range: 4.5-11.0 x 109/L), lymphocyte count of 84x103/mm3 (normal range: 24.0-44.0x103/mm3), hemoglobin 4.2 g/dL (normal range: 13.5-17.5 g/dL), hematocrit 13.9% (normal range 41.0-53.0%), platelets 32,000 µL (normal range: 140,000-440,000 µL), absolute neutrophil count 190 mm3 (normal range: 1300-7800 mm3). The Hepatitis panel and COVID-19 tests were all negative. Physical examination showed conjunctival pallor and splenomegaly extending to the umbilicus.

**Figure 1:**
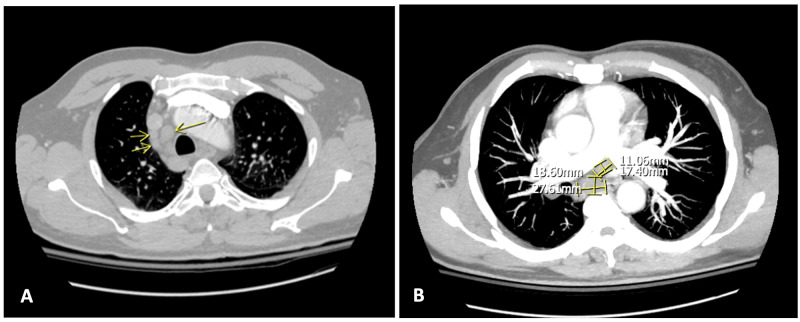
Axial images (A and B) from a CTA demonstrate mediastinal adenopathy with the largest node measuring 27.6x18.6 mm in the subcarinal region. CTA: computer tomography angiography

**Figure 2:**
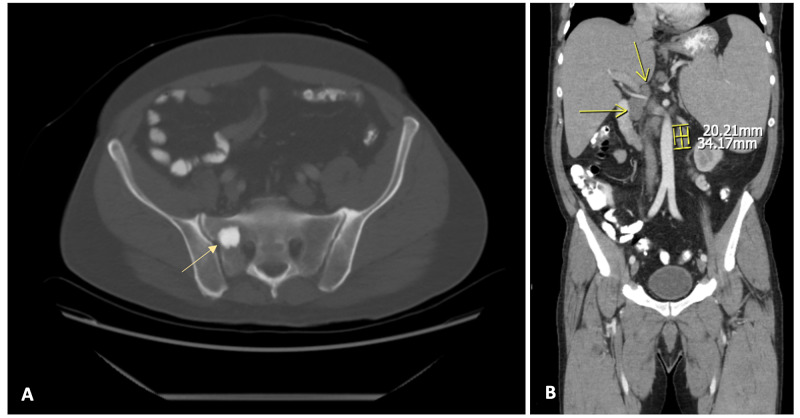
An axial image (A) from a CT pelvis shows an increased signal in the right sacrum and the coronal image (B) showing periportal and retroperitoneal lymph nodes suspicious for lymphoid neoplasm.

Further laboratory investigation revealed a soluble IL-2 receptor level >20,000 pg/mL (normal range 622-1619 pg/mL). Multiple myeloma workup was negative. Imaging revealed splenomegaly measuring 25cm on ultrasonography. Computer tomography angiography (CTA) of the chest showed mediastinal adenopathy (Figure 1), and computer tomography of the pelvis showed an increased signal in the right sacrum (Figure 2).

**Figure 3:**
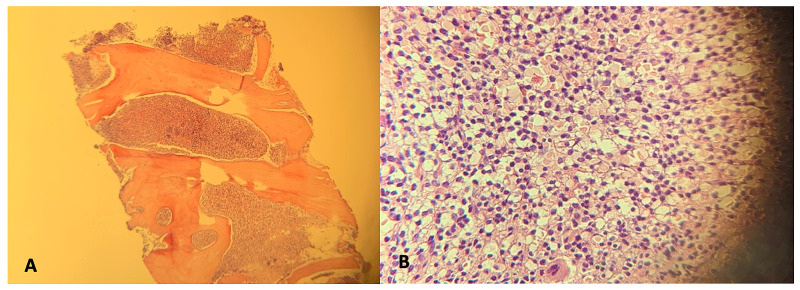
Bone marrow under low magnification (A) demonstrating hypercellularity and medium magnification (B) showing extensive involvement by atypical lymphocytes with abundant cytoplasm and central oval nuclei (fried-egg appearance).

There was no evidence of metastasis on the bone scan. A bone marrow biopsy was performed initially revealing a dry tap. Aspiration was subsequently achieved and analysis showed complete maturation of myeloid and erythroid populations, no megakaryocytes, and no increase in blasts or plasma cells. The marrow was found to be hypercellular with extensive involvement by atypical lymphocytes which had replaced >90% of marrow cellularity. The B-cell population was 18%. Flow cytometry showed a monoclonal population of kappa, CD103, CD25, CD11c, and CD10 positive B-cells. Subsequent testing was positive for BRAF mutation. The bone marrow smear showed small to mildly enlarged atypical lymphoid cells with round to slightly irregular nuclear contour with considerable cytoplasm (Figure 3).

The patient was previously treated with five days of cladribine in Ukraine. As the patient had relapsed seven years after initial treatment, National Comprehensive Cancer Network (NCCN) guidelines dictated that he could be treated again with the same purine analog. We treated the patient with cladribine and concurrent rituximab followed by weekly maintenance rituximab. At the time of this case report submission, the response to treatment has been favorable with near resolution of splenomegaly, improvement in fatigue, and resolution of bone pain.

## Discussion

HCL is a lymphoid malignancy characterized by the increased neoplastic proliferation of small mature B cells with abundant cytoplasm. The cytoplasm has characteristic circumferential, thin, cytoplasmic projections which can be seen in both the bone marrow, peripheral blood, and splenic red pulp. These abnormal hairy cells can become sequestered in the spleen which results in splenomegaly and varying degrees of cytopenia. The cytopenia in HCL can vary from mild to severe and may affect the production of red blood cells, platelets, mature granulocytes, and monocytes. Routine blood work showing anemia, thrombocytopenia, and pancytopenia may initially raise suspicion for HCL in asymptomatic patients while more overt findings such as bleeding or infection may be the presenting signs in symptomatic patients [4]. An article in Blood Research from 2020 compiled atypical presentations of HCL citing case reports of absent splenomegaly, arthralgia, skull lesions, epidural mass with radiculopathy, and a femur lesion [5].

Two types of HCL have been described, the first being classical hairy cell leukemia (HCLc) and the second, variant hairy cell leukemia (HCLv) [6]. HCLv is a chronic B cell lymphoid neoplasm previously thought to be a subtype of HCL but now considered an entity that is distinct from HCLc. HCLv is more aggressive and has a poor response to standard HCL treatment [7].

The initial laboratory evaluation of HCL includes a complete blood count with differential (CBC w/dif), a peripheral blood smear, comprehensive metabolic panel (CMP), electrolytes, uric acid, lactate dehydrogenase (LDH), CD4 count, HIV and hepatitis panel. If HCL is suspected, along with routine labs, a peripheral blood flow should be ordered to confirm the immunophenotyping of circulating mononuclear nuclear cells [6]. A bone marrow aspiration and biopsy should be performed as soon as possible for a definitive diagnosis. The bone marrow aspiration may be a dry tap because of the development of fibrosis. Imaging is not part of the diagnostic criteria for HCL but can be helpful to document splenomegaly and lymphadenopathy.

Most patients with HCL achieve durable remission with purine analog treatment with pentostatin or cladribine and rituximab [8,9]. The use of interferon therapy and splenectomy are no longer recommended treatments [10]. The response to treatment can be monitored with a physical examination of spleen size and a CBC w/dif. A bone marrow biopsy has prognostic value and, although not required, should be done four to six months after cladribine treatment, or after normalization of cell counts on CBC w/dif if treatment is with pentostatin.

The goal of treatment is complete remission (CR). CR is defined as normalization of peripheral blood counts (hemoglobin >11 g/dL), platelets (>100,000/µL), absolute neutrophil count (>1500/µL), absence of morphologic evidence of HCL on both peripheral blood smear and bone marrow smear, and regression of splenomegaly - although radiographic documentation of spleen size is not necessary [6]. Minimal residual disease (MRD) is defined as HCL infiltrates recognizable only using polymerase chain reaction testing but not identifiable with traditional immunohistochemical (IHC) evaluation or with flow cytometry. Depending on the criteria used, 13 to 53 percent of patients in apparent CR have evidence of MRD, which may or may not predict future relapse [11-14]. 

The majority of patients treated with purine analogs will respond; up to 20% will achieve partial remission and up to 4% will be classified as a resistant disease and have stable or progressive disease [15]. Patients with a relapse of HCL should be treated based on the timing of the relapse. Relapse within two years of initial treatment can be treated with an alternative purine analog, while patients who relapse after two years can be treated with the same purine analog used previously and the addition of rituximab [6]. Patients with HCL resistance to two or more therapies are candidates for either moxetumomab pasudotox, vemurafenib with rituximab, or bendamustine with rituximab [16].

Moxetumomab pasudotox is an anti-CD22 antibody that has shown good response rates in patients with previously treated HCL but has not been effective in HCLv. A multicenter study evaluated moxetumomab pasudotox in patients with either relapsed or refractory HCL who had failed treatment with at least two prior therapies, including at least one purine nucleoside analog [17-19]. Administration of this therapy is complicated and requires hydration, premedication, and monitoring for renal toxicity, electrolyte abnormalities, capillary leak syndrome, and hemolytic uremic syndrome. Long-term follow up is still needed to further evaluate the durability of remission and long-term side effects. Moxetumomab pasudotox is currently approved by the US Food and Drug Administration for adults with relapsed or refractory HCL who received at least two prior therapies, including treatment with a purine nucleoside analog [19]. 

## Conclusions

HCL can present with a variety of symptoms ranging from asymptomatic to pancytopenia and massive splenomegaly. A routine CBC is valuable in the detection of HCL, as it is most often asymptomatic at diagnosis. Patients presenting with pancytopenia or cytopenia with splenomegaly should be worked up for HCL. Not all patients with HCL require treatment. Treatment indications include anemia (hemoglobin <10 g/dL), thrombocytopenia (<100,000 µL), neutropenia (WBC >1500/µL), recurrent infections, symptomatic splenomegaly or symptomatic lymphadenopathy. The first-line treatment for HCL includes purine analogs and rituximab with a durable response. Hairy cell Leukemia can have bone lesions and may present with bone pain as we have seen in this case study. A peripheral smear is helpful in the diagnosis of HCL although, as demonstrated here, not all patients will have characteristic hairy cells on peripheral smear. Therefore, IHC is crucial to the diagnosis of HCL. 
